# α-Synuclein Aggregation Is Triggered by Oligomeric Amyloid-β 42 via Heterogeneous Primary Nucleation

**DOI:** 10.1021/jacs.3c03212

**Published:** 2023-08-09

**Authors:** Devkee M. Vadukul, Marcell Papp, Rebecca J. Thrush, Jielei Wang, Yiyun Jin, Paolo Arosio, Francesco A. Aprile

**Affiliations:** † Department of Chemistry, Molecular Sciences Research Hub, Imperial College London, London W12 0BZ, U.K.; ‡ Department of Chemistry and Applied Biosciences, Institute for Chemical and Bioengineering, Swiss Federal Institute of Technology, 8093 Zurich, Switzerland; § Institute of Chemical Biology, Molecular Sciences Research Hub, Imperial College London, London W12 0BZ, U.K.

## Abstract

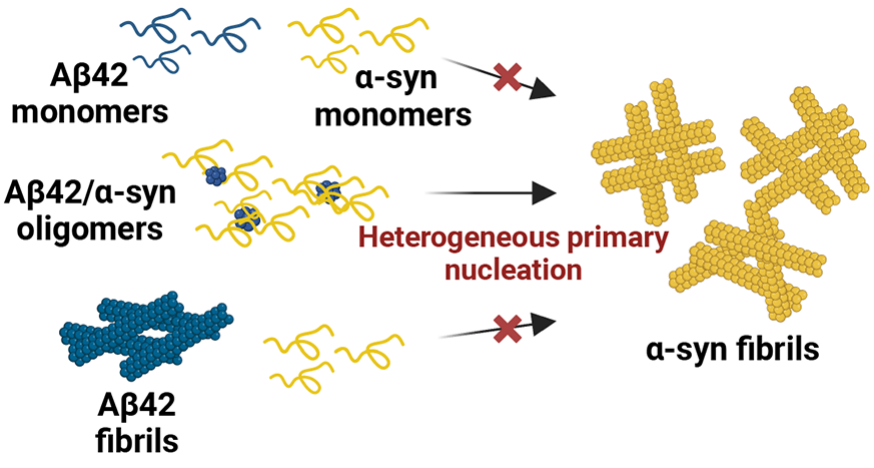

An increasing number of cases where amyloids of different proteins are found in the same patient are being reported. This observation complicates diagnosis and clinical intervention. Amyloids of the amyloid-β peptide or the protein α-synuclein are traditionally considered hallmarks of Alzheimer’s and Parkinson’s diseases, respectively. However, the co-occurrence of amyloids of these proteins has also been reported in patients diagnosed with either disease. Here, we show that soluble species containing amyloid-β can induce the aggregation of α-synuclein. Fibrils formed under these conditions are solely composed of α-synuclein to which amyloid-β can be found associated but not as part of the core of the fibrils. Importantly, by global kinetic analysis, we found that the aggregation of α-synuclein under these conditions occurs via heterogeneous primary nucleation, triggered by soluble aggregates containing amyloid-β.

## Introduction

Neurodegenerative diseases, such as Alzheimer’s (AD) and Parkinson’s (PD) diseases, are characterized by the formation of fibrillar protein aggregates, called amyloids, in the nervous system.^1^ Amyloids are enriched in cross-β sheets and resistant to degradation.^2,3^ In the case of AD, a key pathological hallmark is the extracellular amyloid plaques made of the amyloid-β peptide (Aβ).^4^ Aβ exists as variants of different lengths (i.e., 37–49 residue-long) and originates by the proteolytic cleavage of the amyloid protein precursor.^5−7^ Aβ monomers undergo a self-assembly process that leads to the formation of amyloids via small transient intermediates, called oligomers.^4^ Thus far, Aβ monomers and amyloids are considered less pathogenic compared to the oligomers.^8−11^ Several toxic effects of Aβ oligomers have been identified, including inflammation, synaptotoxicity, membrane permeabilization and oxidative stress.^8,9,12−19^

The co-occurrence of amyloids composed of Aβ and of other proteins has been detected in several neurodegenerative diseases, suggesting an interplay between Aβ and these amyloid forming proteins.^20−26^ One such protein is α-synuclein (α-syn), which is found as amyloid fibrils in the Lewy bodies (LBs) in PD, Lewy body dementia, and multiple system atrophy.^27−29^ The identification of Aβ plaques in up to 50% of PD patients^30,31^ and of LBs in almost 50% of AD patients^32^ suggests a crosstalk between Aβ and α-syn. This hypothesis is further supported by the identification of the non-amyloid-β component (NAC) region of α-syn in AD plaques.^32,33^ Additionally, as Aβ has been found to accumulate intracellularly,^34^ where α-syn is found,^35^ it is likely that the two proteins interact within the cellular environment.

The effects of Aβ and α-syn on each other’s aggregations are not yet fully characterized at the molecular level. Recently, it has been found that α-syn can either accelerate or inhibit the aggregation of Aβ. This dual mechanism of α-syn has been observed on both the 40-residue-long (Aβ40) and the 42-residue-long (Aβ42) isoforms of Aβ.^22,36−40^ Whether α-syn promotes or inhibits Aβ aggregation seems to depend on the aggregated state of α-syn. In fact, it has been shown that α-syn monomers can delay the aggregation of Aβ42, while α-syn amyloid fibrils can accelerate it.^41^ Conversely, it has been shown that Aβ42 is able to trigger the aggregation of α-syn.^42^ Furthermore, the formation of hetero-oligomers made of both Aβ42 and α-syn has been observed *in vitro*, suggesting that the two proteins can coaggregate and form heterogeneous aggregates.^43−45^ However, to date, there is only limited information on the mechanism by which Aβ induces the aggregation of α-syn or the structure of the aggregates, which are formed when the two proteins are present together.

Here, we investigate the structure and the kinetics of formation of aggregates formed when α-syn and Aβ42 coaggregate *in vitro.* We have chosen Aβ42 over Aβ40 for this work as elevated Aβ42 is a key feature of AD.^46^ We report that the aggregates formed when both proteins are coincubated are α-syn fibrils whose surface is coated by Aβ42. The mechanism of formation of these fibrils can be described with a model where oligomeric Aβ42 provides α-syn monomers with a surface to nucleate, similarly to what has been seen with lipids.^47^ Our results provide a first mechanistic understanding on how these two key amyloidogenic proteins can possibly interact when found coaggregated in patients. They also reveal a new toxic mechanism of Aβ42, namely, its ability to trigger the formation of amyloids of other proteins.

## Materials and Methods

### Expression and Purification of Aβ42

Purification of Aβ42 was carried out as previously described.^48^ Briefly, the Aβ42 peptide conjugated with the spider silk domain (known as the fusion protein, 20 kDa) was expressed by heat-shock transformation in BL21 *Escherichia coli* (*E. coli*). Cells were grown in Luria−Bertani broth supplemented with kanamycin (50 μg/mL) at 37 °C with shaking at 200 rpm in a New Brunswick Innova 44R Incubator Shaker (Eppendorf, Hamburg, Germany) until they reached an OD of 0.8 and were then induced overnight with with 1 mM IPTG at 20 °C with shaking at 200 rpm. Cells were collected the following day by centrifugation, and the pellet was resuspended in 20 mM Tris–HCl and 8 M urea, pH 8. The resuspended cells were sonicated on ice for 20 min (15 s on and 45 s off pulses, 20% amplitude) and centrifuged once more to clear cellular debris. The supernatant was filtered using a 0.22 μm filter and loaded onto two HisTrap HP 5 mL columns (Cytiva, Little Chalfont, UK) in tandem that had been pre-equilibrated with 20 mM Tris–HCl and 8 M urea, pH 8 supplemented with 15 mM imidazole (binding buffer). Following sample application, the columns were washed with several column volumes of binding buffer. The fusion protein was then eluted with 5 column volumes of 20 mM Tris–HCl and 8 M urea, pH 8 supplemented with 300 mM imidazole (elution buffer). This was then collected and dialyzed overnight against 20 mM Tris–HCl, pH 8. After dialysis, the concentration of the fusion protein was measured using a Nanodrop. TEV protease was added to the fusion protein at a 1:15 molar ratio overnight at 4 °C. Following this, 7 M guanidine-HCl was added to the sample and incubated on ice for at least 2 h before applying the sample on to a Superdex 75 Increase pg 10/600 column (Cytiva, Little Chalfont, UK) pre-equilibrated with 20 mM phosphate buffer supplemented with 200 μM EDTA, pH 8 for size-exclusion chromatography. Peaks were collected manually. The concentration of monomeric Aβ42 (in μM) was determined from the chromatogram using the following calculation:
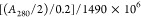
where A_280_ is the absorbance at 280 nm of the elution peak of Aβ42, 0.2 is the path length (cm) of the ATKA Pure (Cytiva, UK) and 1490 M^–1^ cm^–1^ is the molecular coefficient of Aβ42.

The stock concentration was diluted to 2 μM for experiments in 20 mM phosphate buffer supplemented with 200 μM EDTA, pH 8. For Aβ42 fibrils, monomeric Aβ42 was incubated for 24 h after size exclusion at 37 °C. The fibrils were collected by centrifugation at max speed for 30 min after which the concentration of the supernatant was measured with a Nanodrop (Thermo Fisher Scientific, Waltham, MA, USA). The concentration of the supernatant was subtracted from the initial monomeric Aβ42 concentration to obtain the fibril concentration. These were then diluted to 2 μM for experiments in 20 mM phosphate buffer supplemented with 200 μM EDTA.

### Expression and Purification of Monomeric α-Syn, and Preparation of α-Syn Fibrils

The pT7-7 α-syn WT plasmid (a gift from Hilal Lashuel, Addgene, Watertown, NY, United States^49^), with codon Y136 mutated from TAT to TAC to prevent cysteine misincorporation, was transformed into BL21-Gold (DE3) competent *E. coli* (Agilent Technologies, Santa Clara, CA, USA) according to the manufacturer’s instructions. Expression of α-syn was induced using 1 mM IPTG at 28 °C overnight, and ampicillin (100 μg/mL) was included as necessary. The cells were harvested by centrifugation and resuspended in 20 mM Tris−HCl and 1 mM EDTA, pH 8.0 and protease inhibitors (Roche, Basel, Switzerland). One protease inhibitor tablet was added per litre of culture. α-Syn was then purified as previously described.^50^ Anion exchange chromatography was performed using a HiPrep Q HP 16/10 column (Cytiva, Little Chalfont, UK) and a linear gradient from 0 to 1 M NaCl. Finally, monomeric α-syn was purified by size exclusion chromatography in PBS (137 mM NaCl, 2.7 mM KCl, 10 mM Na_2_HPO_4_, and 1.8 mM KH_2_PO_4_, pH 7.4) using a HiLoad 16/600 Superdex 75 pg (GE Healthcare, UK). The concentration of the protein was determined by measuring the absorbance at 275 nm, using an extinction coefficient of 5600 M^−1^ cm^−1^. α-Syn fibrils were obtained by incubating 60 μM α-syn at 37 °C under quiescent conditions for two weeks. NaN_3_ (0.02%) was added to each sample to prevent the growth of bacteria.

### Thioflavin T Fluorescence Assays

Monomeric α-syn (20−140 μM) and monomeric or fibrillar Aβ42 (2−8 μM) were incubated alone or together in 20 mM phosphate buffer, 200 μM EDTA, pH 8, ensuring that the final ionic strength did not exceed 96 mM. Aggregations in low phosphate buffer were performed in 4 mM phosphate buffer, 200 μM EDTA, pH 8. Samples were prepared with a final concentration of 10 μM thioflavin T (ThT) dye, gently vortexed, and pipetted into nonbinding surface black 96-well plates (Greiner Bio-One, Frickenhausen, Austria) in triplicates. The plate was read in a ClarioStar Plus microplate reader (BMG LabTech, Ortenberg, Germany) at 37 °C. The excitation and emission wavelengths were set to 440 and 480 nm, respectively, and fluorescence intensity measurements were taken using spiral averaging (3 mm diameter). Buffer-only values were subtracted from the sample readings. Readings were taken every 2–5 min. The data were plotted using GraphPad Prism version 9.3.1 for Windows (GraphPad Software, San Diego, CA, United States).

### Immunogold Labeling and Negative Stain Transmission Electron Microscopy

Samples were prepared and incubated for 24 h at 37 °C before 4 μL was spotted onto Formvar/carbon-coated 300 mesh copper grids for 1 min. Excess sample was removed by blotting dry with Whatman filter paper, and the grid was allowed to dry for 2 min. Samples for negative stain transmission electron microscopy (TEM) were then washed with 4 μL of water and stained with 4 μL of 2% w/v uranyl acetate. Samples for immunogold labeling were blocked using normal goat serum—1:10 dilution in PBS+ [1% BSA, 500 mL/L Tween-20, 10 mM Na EDTA, and 0.2 g/L NaN_3_] for 15 min after which the grids were incubated on a 20 μL drop of primary antibodies—1:10 dilution of 6E10 (Biolegend, San Diego, CA, USA), and anti-α-syn MJFR1 (Abcam, Cambridge, UK) in PBS at room temperature for 2 h. The grids were then washed with PBS+ three times for 2 min and incubated on a 20 μL drop of secondary antibodies conjugated with gold particles (1:20 dilution of antimouse and antirabbit secondary antibodies conjugated with 10 and 6 nm gold particles, respectively, Abcam, Cambridge, UK). Finally, the grids were washed five times with PBS+ and five times with water for 2 min before being stained with 2% w/v uranyl acetate. Grids were imaged on a T12 Spirit electron microscope (Thermo Fisher Scientific (FEI), Hillsboro, OR, USA). The fibril width was measured using Fiji. All data were plotted using GraphPad Prism version 9.3.1 for Windows (GraphPad Software, San Diego, CA, United States). Gold particles (6 and 10 nm) were assigned colors (yellow and blue, respectively) after imaging using an in-house Python script using the morphology module of SKimage.

### Dot Blotting

Dot blots were carried out on samples that were aggregated in the microplates without ThT at the endpoint of aggregation. Samples were collected and centrifuged at max speed (∼17,000*g*) for 30 min on a benchtop centrifuge to separate the soluble and insoluble aggregates. Three to five repeats of each sample were spotted onto a 0.45 μM nitrocellulose membrane and blocked in 5% nonfat milk in 0.1% PBS-Tween for 1 h at RT. The membranes were then incubated in primary antibodies (1:1000 dilution in 0.1% PBS-Tween for both anti-α-syn and 6E10) overnight at 4 °C under constant shaking. The following day, the membranes were washed three times for 10 min each in 0.1% PBS-Tween. Membranes were then incubated in secondary antibodies conjugated with an AlexaFluor tag (anti-mouse 647 and anti-rabbit 647, diluted with 1:2000 and 1:5000 0.1% PBS-Tween, respectively, Thermo Fisher Scientific, Waltham, MA, USA) at room temperature for 1 h, protected against light. Following three further washes for 10 min each in 0.1% PBS-Tween, the membranes were detected with the appropriate laser using a Typhoon scanner (GE Healthcare, Amersham, UK).

### PK Digestion

Fibrils were collected from the coincubation sample at the end of aggregation by centrifugation at max speed (∼16,000*g*) for 1 h. α-Syn fibrils were collected after two weeks of incubation at 37 °C under quiescent conditions. Fibrils were resuspended in buffer and treated with 0–15 μg/mL PK for 20 min. Samples were then incubated at 95 °C for 5 min to stop the enzymatic reaction, and samples were prepared for SDS-PAGE and Western blotting analysis. The primary antibodies used for this analysis are detailed in Table S1 and were purchased as a kit from Cosmo Bio Co., Ltd. (Japan, Tokyo). Densitometry was carried out on the putative monomeric band using Fiji software, and the IC_50_ was calculated from normalized data using the GraphPad Prism (GraphPad Software, CA, USA).

### SDS-PAGE and Western Blotting

Fibrils from the coincubation sample were collected by centrifugation at max speed for 30 min, and the supernatant (S1) was removed and separated from the pellet (P1). The pellet was washed with 50 μL of 2% SDS and centrifuged. The supernatant was again removed (S2), and the pellet was resuspended in 50 μL of buffer to remove any residual SDS (P2); the pellet was centrifuged, and the supernatant (S3) and pellet were separated (P3) after which the pellet was treated with 50 μL of 4 M guanidine hydrochloric acid (G-HCl) for 2 h at RT before SDS-PAGE and Western blot analysis. Samples were prepared in 4× LDS sample buffer and 10× reducing agent after which they were boiled at 95 °C for 5 min. Samples were then run on 4–12% Bis-Tris NuPAGE gels (Thermo Fisher Scientific, Waltham, MA, USA) and transferred onto a 0.45 μm nitrocellulose membrane for 7 min at 20 V with the iBlot 2 (Thermo Fisher, Waltham, MA, USA). Blocking, incubation with antibodies, and detections were carried out as described above.

### Native PAGE and Western Blotting

Samples were prepared and aggregated in 96-well microplates with a nonbinding surface for 24 h after which 20 μL of each sample was prepared in native sample buffer and run on Novex Tris-Glycine gels (Thermo Fisher Scientific, Waltham, MA, USA) in native running buffer as per the manufacturer’s instructions. The gel was then transferred onto a 0.45 μM nitrocellulose membrane using an iBlot 2 (Thermo Fisher Scientific, Waltham, MA, USA) for 7 min at 25 V. The membrane was then blocked, incubated with primary and secondary antibodies, and imaged as described above.

## Results and Discussion

### Fibrils Formed in α-Syn–Aβ42 Coincubation Are Made of α-Syn

**Figure 1:**
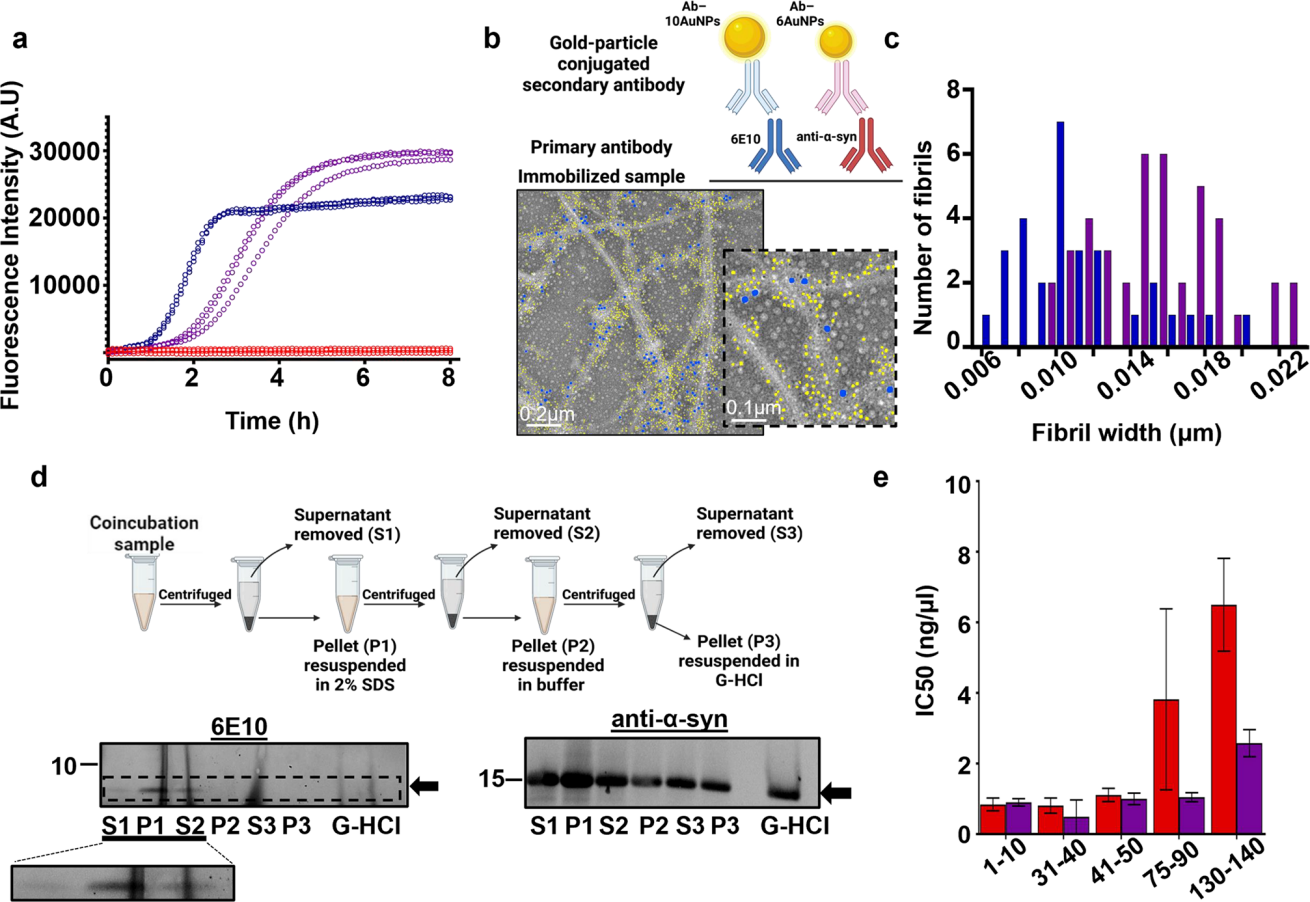
Aggregation of α-syn–Aβ42 coincubation results in formation of α-syn fibrils. (a) ThT fluorescence assay of 60 μM α-syn (red), 2 μM Aβ42 (blue), and α-syn aggregated with Aβ42 (purple). Three technical replicates are shown per condition. (b) Immunogold labeling and negative stain electron micrograph of α-syn aggregated with Aβ42 at the endpoint of aggregation. Fibrils are highly decorated with 6 nm gold particles (Ab–6AuNPs, yellow) specific for α-syn as opposed to the 10 nm gold particles labeling Aβ (Ab–10AuNPs, blue), which are sparse. Yellow and blue dots are represented as 1.5× their actual size for clarity. (c) Width distribution of Aβ42 fibrils (blue, *n* = 32) and fibrils in the coincubation sample (purple, *n* = 32). (d) Schematic of G-HCl treatment of the SDS insoluble fraction (top). Fibrils from the coincubated sample were collected by centrifugation, and the supernatant (S1) was removed and separated from the pellet (P1). The pellet was washed with 2% SDS and centrifuged. The supernatant was again removed (S2), and the pellet was resuspended in buffer (P2). The pellet was centrifuged, and the supernatant (S3) and pellet were separated (P3) after which the pellet was treated with 4 M G-HCl for 2 h at RT before SDS-PAGE and Western blot analysis. Detection with the 6E10 antibody (bottom left) revealed no Aβ42 in the G-HCl-treated pellet; however, α-syn was detected with the anti-α-syn antibody (bottom right). Monomers of the proteins are indicated by the black arrows. (e) Proteinase K digestion of α-syn fibrils formed in the absence (red) and presence (purple) of Aβ42 quantified by densitometry and SDS-PAGE and Western blotting. Quantification from two independent experiments. The error bars are representative of the standard error of the mean.

To assess the effects of Aβ42 on α-syn aggregation, we carried out ThT assays on solutions containing 60 μM monomeric α-syn in the presence of a substoichiometric concentration (2 μM) of monomeric Aβ42 (Figure 1a and Figure S1a–c). Hereafter, we will refer to this condition as the coincubation sample. As controls, we also performed ThT experiments on solutions containing either 60 μM α-syn or 2 μM Aβ42. We found that α-syn alone did not aggregate during the time of this experiment (8 h), while Aβ42 alone aggregated in a timescale of approximately 4 h as previously reported.^48^ In the coincubation sample, we observed an increase of the ThT signal, which was slower than the aggregation profile of Aβ42 alone. In fact, the aggregation half-time (*t*_50_) of the coincubation sample was approximately 1.5-fold the one of Aβ42 alone (3 ± 0.4 and 2 ± 0.4 h, respectively) (Figure S1d). Furthermore, by negative stain TEM, we confirmed that the aggregates formed in the coincubation had a fibrillar conformation (Figure S1d).

To investigate the protein composition of the fibrils from the coincubation sample, we performed immunogold TEM on samples collected at the aggregation endpoint (24 h) (Figure 1b and Figure S2). To do so, the samples were simultaneously probed for Aβ42 with antibodies conjugated to 10 nm gold nanoparticles (Ab–10AuNPs, blue) and α-syn with antibodies conjugated to 6 nm gold nanoparticles (Ab–6AuNPs, yellow). We found no or extremely low signal for Aβ42 in the sample with α-syn alone and for α-syn in the samples with Aβ42 alone, verifying that our staining is specific (Figure S2). The sample containing α-syn alone showed a diffuse distribution of small clusters of Ab–6AuNPs, confirming the presence of soluble α-syn and the lack of fibrils (Figure S2c), similarly to a previous report^42^ and in agreement with our ThT and negative staining TEM data. The sample with Aβ42 only contained fibrils decorated by Ab–10AuNPs, i.e., made of Aβ42 (Figure S2a). While these controls confirm the specificity of our labeling protocol, there is seemingly less labeling of Aβ42 compared to α-syn. This could be due to a lower solvent accessibility of the antibody epitope in Aβ42 fibrils as compared to α-syn fibrils.^51,52^ Fibrils were also present in the coincubation sample (Figure 1b). However, these fibrils were structurally distinct from Aβ42 fibrils. The median of the widths of the fibrils from coincubation (16 nm) was larger than the one of Aβ42 fibrils (10 nm) (Figure 1c). Furthermore, the fibrils in the coincubation sample were predominantly decorated by 6Ab–AuNPs, i.e., made of α-syn. We also compared the fibrils in the coincubation sample to α-syn fibrils formed in quiescent conditions at 37 °C (Figure S3). Our analysis shows that these fibrils are similar compared to the fibrils made in the coincubation sample (median width of 16 nm).

From the immunolabeling, some Ab–10AuNPs could also be detected on these fibrils. To understand whether Aβ42 was peripheral or within the SDS-insoluble core of the fibrils, we quantified Aβ42 and α-syn in the SDS-insoluble protein fraction at the aggregation endpoint. To do so, we washed the fibrils with SDS to remove any associated soluble aggregates and then treated the fibrils with 4 M guanidine hydrochloric acid (G-HCl). This was then analyzed by SDS-PAGE and Western blotting (Figure 1d). We found that only α-syn was in the SDS-insoluble fractions, indicating that the core of the fibrils from the coincubation sample is composed of α-syn only, and Aβ42 is on the surface but not within the core of the fibrils.

To further investigate the stability of the coincubation fibrils, we treated them with varying concentrations of proteinase K (PK). We then measured the PK-resistant α-syn species by SDS-PAGE and Western blotting as well as densitometry using an array of antibodies scanning the sequence of α-syn (Table S1). Based on the densitometric analysis of the putative monomer, we found that the PK stability profile of the fibrils in the coincubation sample was largely comparable to the one of fibrils obtained by incubating α-syn for two weeks. However, based on the profile of degraded protein below the monomer band, the fibrils formed from α-syn alone have a more resistant NAC region (probed with the antibody recognizing amino acids 75–90), which makes up the core of α-syn fibrils (Figure 1e and Figure S4). This analysis further confirms the amyloid nature of the fibrils in the coincubation sample and indicates that α-syn fibrils formed in the presence of Aβ42 are structurally distinct to α-syn fibrils formed in the absence of Aβ42.

### α-Syn Aggregation in α-Syn–Aβ42 Coincubation Is Triggered by Oligomeric Aβ42

We then aimed to identify the conformations of Aβ42 that trigger α-syn aggregation.

**Figure 2:**
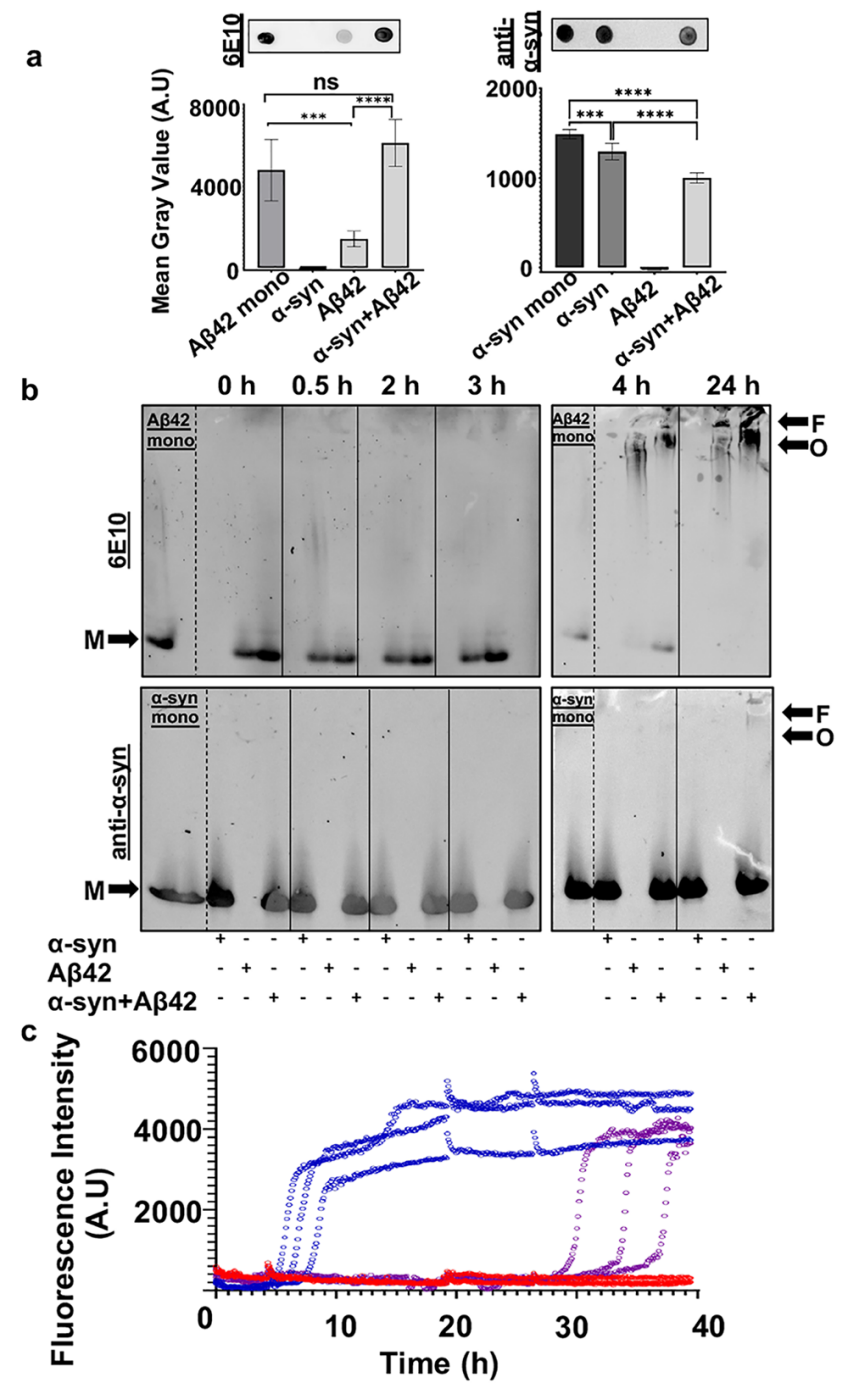
Oligomeric Aβ42-triggered α-syn aggregation. (a) Dot blot analysis and quantifications on the soluble fractions of aggregated samples detected with 6E10 (left) and anti-α-syn (right) primary antibodies. Samples were compared to monomeric protein at the start of aggregation. Five repeats for each sample were quantified. The error bars represent the standard deviation. Mean gray values were compared with one-way ANOVA and Tukey’s multiple comparison test where ns *p* > 0.05, ***p ≤ 0.001, ****p ≤ 0.0001. (b) Native PAGE and Western blot analysis of total samples during aggregation. Detection with the 6E10 antibody (top) and anti-α-syn (bottom). Our results show that high-molecular-weight assemblies of α-syn, O (putative oligomers) and F (putative fibrils) are not seen until oligomers of Aβ42 are formed at 4 h, as compared to the monomers of both proteins (M). (c) ThT fluorescence assay of 2 μM Aβ42 (blue), 60 μM α-syn (red), and the coincubation sample (purple) in low phosphate buffer (4 mM). Three individual technical replicates are shown.

We performed immuno-dot blots on soluble protein fractions at the endpoint (24 h) of aggregation and compared this to monomeric protein at the start of aggregation (Figure 2a and Figure S4). As expected, we observed that soluble Aβ42 drastically decreases (by ∼70%) when incubated alone. On the contrary, in the coincubation sample, the soluble protein species does not significantly change. Soluble α-syn decreases by only ∼10% when the protein is incubated alone, while, in the coincubation sample, it reduces by ∼30%. By native PAGE and Western blotting on protein total fractions (Figure 2b), we found that, during aggregation, Aβ42 alone progressively forms protein species that do not enter the gel, which is a feature of fibrils. Instead, α-syn alone remains mainly monomeric. In the coincubation sample, more Aβ42 can enter the gel as compared to the Aβ42 alone condition. In particular, the protein forms high-molecular-weight oligomers after 4 h of incubation. Additionally, high-molecular-weight assemblies of α-syn are not detected until Aβ42 oligomers are formed. Together, these data indicate that oligomeric Aβ42 promotes α-syn aggregation.

To understand whether monomeric or aggregated Aβ42 is responsible for triggering α-syn aggregation, we performed ThT experiments under conditions where Aβ42 aggregation is delayed. Our hypothesis was that if Aβ42 aggregated forms, but not the monomers, are responsible for triggering α-syn aggregation, then conditions that delay the aggregation of Aβ42 also delay α-syn aggregation when the two proteins are coincubated. Thus, we performed ThT experiments in 4 mM phosphate buffer instead of 20 mM phosphate buffer (Figure 2c). We found that α-syn does not aggregate in low phosphate conditions similarly to when it is incubated in 20 mM phosphate buffer. Aβ42 aggregates significantly slower in 4 mM phosphate buffer compared to 20 mM phosphate buffer. In coincubation conditions, α-syn aggregation is also significantly delayed, suggesting that a direct interaction between α-syn and aggregated, but not monomeric, Aβ42 plays a key role in the process.

**Figure 3:**
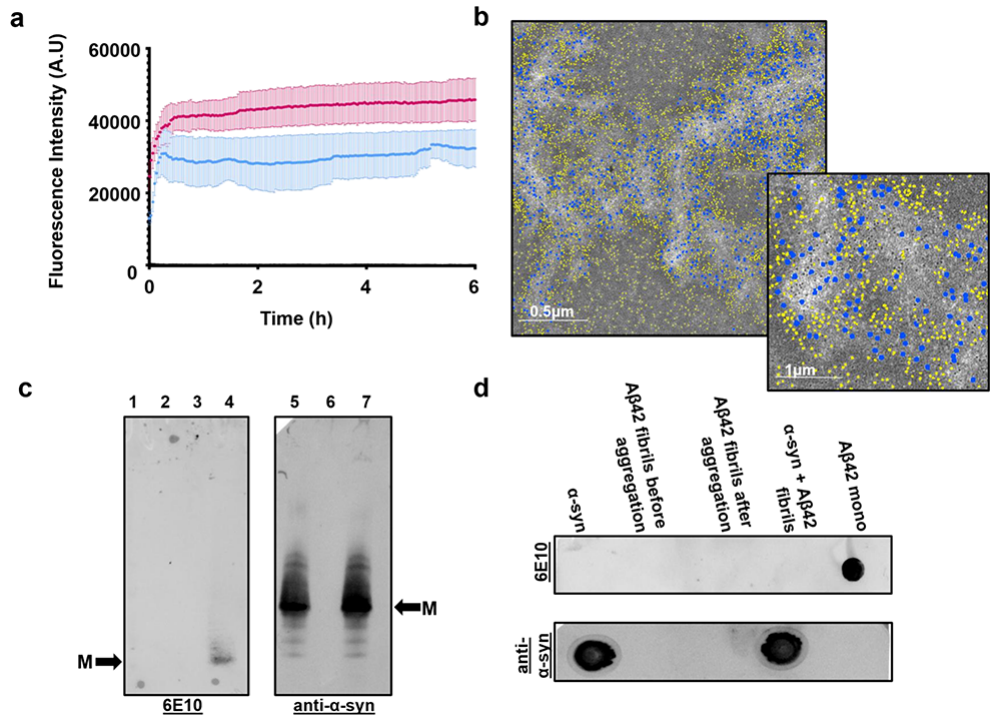
Aβ42 fibrils do not trigger the aggregation of α-syn. (a) ThT fluorescence assay of 60 μM α-syn (black), 2 μM Aβ fibrils (blue), and α-syn aggregated with Aβ fibrils (pink). The average of two technical replicates for each condition is shown. The error bars represent the standard deviation. (b) Immunogold labeling and negative stain TEM of α-syn aggregated with Aβ fibrils and Aβ fibrils alone at the endpoint of aggregation. Fibrils are highly decorated with 10 nm gold particles (Ab–6AuNPs, blue) specific for Aβ42 as opposed to 6 nm gold particles labeling α-syn (Ab–10AuNPs, yellow), which are largely in the surrounding area of the fibrils. Yellow and blue dots are represented as 1.5× their actual size for clarity. (c) Native PAGE and Western blot analysis of total samples after aggregation. Detection with 6E10 (left) revealed no detection of (1) α-syn, (2) that Aβ42 fibrils alone do not enter the gel, and (3) the coincubated sample also has no detectable Aβ42 in the gel; however, (4) freshly purified Aβ42 monomers are detectable. Detection with the anti-α-syn (right) revealed no difference in the molecular weight assemblies of α-syn either (5) alone or (7) aggregated with Aβ42 fibrils. (6) Aβ42 fibrils are not detected by this antibody. M indicates the migration of the putative monomers. (d) Dot blot analysis on the soluble fractions of aggregated samples detected with 6E10 (top) and anti-α-syn (bottom) primary antibodies. No soluble Aβ42 was detected in any sample except freshly purified Aβ42 monomers as expected, and similar intensities of α-syn were detected in the α-syn-only and α-syn aggregated with Aβ42 fibrils samples.

To rule out the possibility that Aβ42 fibrils can also induce α-syn aggregation, we monitored the aggregation that occurs when Aβ42 fibrils are coincubated with α-syn (Figure 3 and Figure S6). Aβ42 fibrils were obtained by aggregating monomeric Aβ42 under quiescent conditions at 37 °C for 24 h. Fibrils were collected by centrifugation and washed with buffer to remove any soluble protein. We measured the ThT fluorescence of samples containing α-syn alone, Aβ42 fibrils alone, and α-syn in the presence of Aβ42 fibrils (Figure 3a). As expected, we observed that α-syn alone does not aggregate. Aβ42 fibrils alone have a steady ThT fluorescence as does the coincubation sample. Immunogold TEM showed that the fibrils in the coincubation sample are largely labeled with the Ab–10AuNPs specific for Aβ (Figure 3b and Figure S6a). This result confirms that there is no aggregation of α-syn and, conversely, that Aβ42 fibrils are not affected by soluble α-syn. Native PAGE and Western blotting on the endpoints of aggregation (Figure 3c) revealed no soluble Aβ42 in the Aβ42 alone and coincubation samples. No difference in the molecular weight of α-syn was observed in the α-syn alone and coincubation samples. This evidence indicates that fibrillar Aβ42 does not affect the aggregation of α-syn or vice versa. Strengthening this observation, dot blot analysis on the soluble and insoluble protein fractions revealed that no soluble Aβ42 was present in the sample containing Aβ42 fibrils alone (Figure 3d and Figure S5b). As expected, soluble Aβ42 was not detected in the coincubation sample, confirming that α-syn had no destabilizing effect on fibrillar Aβ42. Additionally, no difference in the amount of soluble α-syn between the coincubation and α-syn-alone samples was detected.

Based on these data, we rationalize that Aβ42 needs to be able to form oligomers to trigger α-syn aggregation.

### α-Syn Aggregation in α-Syn–Aβ42 Coincubation Occurs via Heterogeneous Primary Nucleation

**Figure 4:**
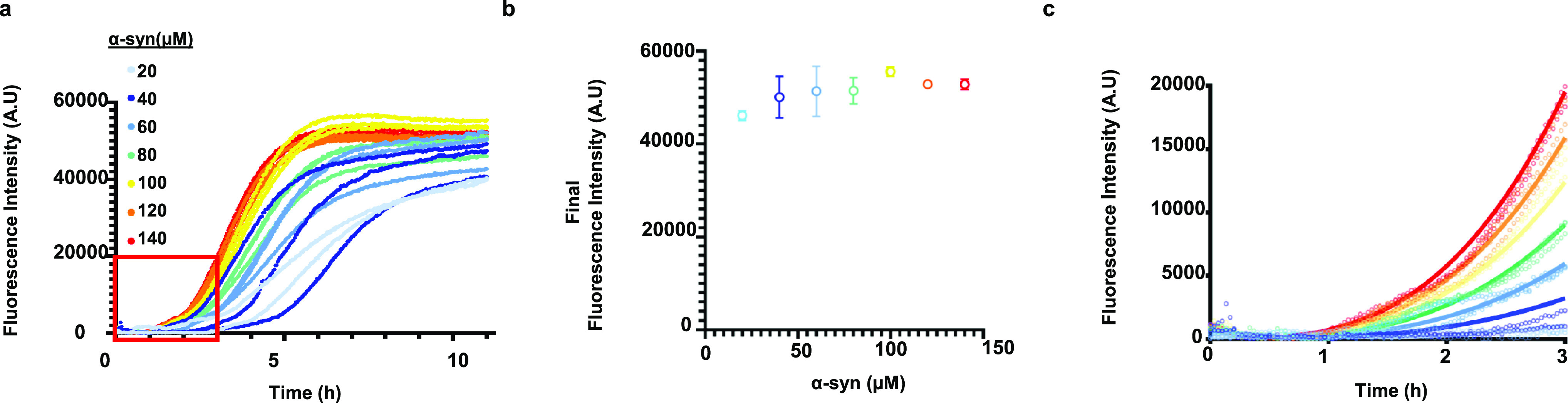
ThT fluorescence measurement of α-syn aggregation in α-syn–Aβ42 coincubation. (a) ThT fluorescence kinetics of 20–140 μM α-syn and 2 μM Aβ42. The data collected in the first 3 h were considered for global fitting, as indicated by the red box. (b) Final ThT fluorescence intensities of samples containing 20−140 μM α-syn and 2 μM Aβ42. The error bars represent the standard deviation. (c) Global fitting of α-syn aggregation. Model parameters *C*, (*n* + o_2_), and *K*M were fitted globally on experimental ThT fluorescence data based on eq S8. The fitted parameters are *C* = 2.27 × 1010, (*n* + o_2_) = 0.69, and *K*M = 230 μM.

The data discussed so far indicate that oligomeric Aβ42 triggers the aggregation of α-syn. To further investigate this aggregation mechanism, we performed ThT fluorescence experiments on solutions containing increasing initial concentrations of monomeric α-syn (ranging from 20 to 140 μM) and 2 μM Aβ42 (Figure 4a).

We found that the final ThT fluorescence intensities in the plateau phase do not increase proportionally with the initial α-syn concentrations (Figure 4b), illustrating that not all monomeric α-syn is converted into fibrils, as observed in our immunoblotting analysis (Figure 2a,b) We further examined this by measuring the concentration of insoluble α-syn at the end of aggregation (∼60 h) when 2 μM Aβ42 was incubated in the presence of 60 and 140 μM α-syn (Figure S7). To do so, soluble α-syn was separated from insoluble α-syn by centrifugation. The concentration of insoluble α-syn was then calculated by subtracting the concentration of soluble α-syn at the end of aggregation from the initial monomer concentration (*t*_0_). We found that there was no significant difference in the amount of insoluble α-syn in both conditions at the end of aggregation (17 and 21 μM, respectively).

In the presence of secondary processes, new nuclei could also form via fibril-dependent nucleation or fragmentation in addition to primary nucleation. In this case, the total amount of fibrils is expected to increase linearly with the initial concentration of α-syn^47^ as the formation of nuclei and hence monomer depletion until complete consumption is not limited during aggregation. However, the constant final amount of fibrils formed at increasing initial monomeric α-syn suggests that, most likely, secondary nucleation processes do not play a substantial role in α-syn aggregation under these conditions. We have confirmed that is due to the complete consumption of Aβ42, which are nuclei, by aggregating 140 μM α-syn with 4 and 8 μM Aβ42 (Figure S8). These data show that the final ThT fluorescence increases as a function of the initial concentration of the Aβ42 monomer.

The lack of secondary processes under our conditions is further supported by the early-time behavior of the kinetic curves: if secondary nucleation or fragmentation was dominant during aggregation, an exponential increase in the amount of fibrillar α-syn would be expected.^53^ However, the ThT fluorescence changes polynomially in time (Figure S9), which indicates that most likely, primary nucleation is the main source of nuclei.^54^

Additionally, we performed native PAGE analysis and Western blotting on the total protein fraction of the coincubation samples at the end of aggregation. We found that Aβ42 remained as high-molecular-weight soluble species that were able to enter the gel in the presence of each of these α-syn concentrations (Figure S10). This result, combined with the observation that neither monomers or fibrils of Aβ42 induce α-syn aggregation, strongly suggests that heterogeneous rather than homogeneous primary nucleation is responsible for the generation of new nuclei. Aβ42 is known to be converted into low-molecular-weight oligomers;^55^ moreover, it was also reported that α-syn can undergo co-oligomerization with Aβ42 to form hetero-oligomeric species.^43^ In our case, these oligomers may provide the surface that triggers the aggregation of monomeric α-syn.

To validate our hypothesis, we applied kinetic modeling to describe heterogeneous primary nucleation of α-syn on the surface of the oligomeric species that are generated during the early times of aggregation.^53−56^ Following the approach of Galvagnion *et al.*,^47^ we limit our analysis to the first 3 h of aggregation where α-syn and Aβ42 monomer concentrations can be approximated as constant, which facilitates analytical description of aggregation. We describe the generation of oligomers and amyloid aggregation subsequent to α-syn binding to the oligomer surface with differential rate laws. For more details of the mathematical model, see supplementary methods.

Global fitting of the model parameters on eq S8 (Figure 4c) shows that our kinetic model is consistent with the evolution of the fluorescence signal at the early times of aggregation. We find a weak dependence of oligomer formation and nucleation on monomeric α-syn with an apparent reaction order of 0.69 (*o*_2_ + *n* in eq S8 in Materials and Methods), which supports our hypothesis on the heterogeneous nature of primary nucleation.^56^ We observed saturation of elongation with a Michaelis–Menten-like constant of *K*_M_ = 230 μM, which is in the same range of with previously reported *K*_M_ values of 46–380 μM for lipid vesicles.^47,57^

## Conclusions

Understanding protein coaggregation is crucial for developing diagnostic and therapeutic strategies for neurodegenerative diseases. Recently, the identification of LBs in up to 50% of AD patients and Aβ plaques in up to 50% of PD patients has led to research interest on the heterogeneous aggregation of these two peptides.

**Figure 5:**
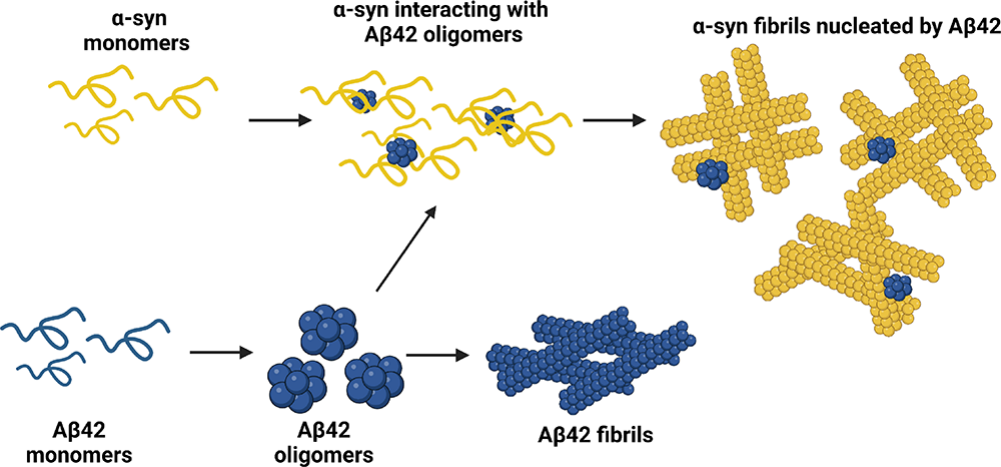
Proposed model for the coaggregation of Aβ42 and α-syn. When in isolation, Aβ42 undergoes a complete aggregation process that leads to the formation of mature amyloid fibrils. In the presence of α-syn, Aβ42 fibril formation is inhibited. We speculate that soluble oligomers of Aβ42, possibly containing α-syn, can promote α-syn aggregation via heterogeneous primary nucleation.

Although it has previously been reported that α-syn aggregation is triggered by Aβ42^42^ and that Aβ42 aggregation is inhibited by α-syn,^39^ our findings unify the mechanism of this coaggregation, which has previously remained elusive (Figure 5). It has been shown by Köppen *et al*. that α-syn has a significantly higher propensity to aggregate when in the presence of low concentrations of Aβ42.^42^ Our data both support this finding and expand on the mechanism by which this occurs. We report that when Aβ42 and α-syn are coincubated *in vitro*, homogeneous amyloid fibrils of α-syn are formed. We also show that under these conditions, Aβ42 is associated to but not part of the core of α-syn fibrils and mainly in the oligomeric conformation. This is in line with the findings of Chau and Kim who reported that soluble α-syn species promoted the formation of stabilized Aβ42 oligomers.^39^ Additionally, we observed that, under these conditions, α-syn aggregates via heterogeneous primary nucleation. Of note, Aβ42 and α-syn hetero-oligomers have also been previously identified.^43−45^ As fibrils composed of both proteins have not been identified, based on our data, we speculate that oligomers of Aβ42, possibly containing α-syn, can serve as nucleation sites for the formation of homogeneous α-syn fibrils. The mechanism observed in this work shares remarkable features of α-syn aggregation in the presence of lipid membranes,^47^ which has also shown to occur via heterogeneous primary nucleation, suggesting that this could be a global property of α-syn when there is a suitable surface to trigger its aggregation. While we speculate that the mechanism of α-syn aggregation is likely to be similar in the presence of several biomolecules, the morphology of the aggregates formed is likely to be distinct. This in line with the identification of several α-syn fibril polymorphs. The physicochemical parameters of these suitable surfaces remain unclear, although hydrophobicity is likely to play an important role, considering also that the air/water interface is a well-known trigger of α-syn aggregation.^58^

It is important to note that α-syn in bulk is not highly aggregation-prone. In fact, in general, the protein requires the interaction with other biomolecules to aggregate.^47,59−62^ Therefore, Aβ42-containing oligomers triggering α-syn aggregation are not surprising, and a similar stoichiometric relationship has been reported for α-syn and exosomes^63^ and glycosaminoglycans.^64^

It is plausible that the interaction of α-syn and Aβ42 drives Aβ42 to form highly stable, off-pathway oligomers that could have additional toxic effects besides triggering α-syn aggregation.^65^ We believe that triggering the aggregation of α-syn could be regarded as an additional toxic mechanism of Aβ42. While oligomers of Aβ are widely accepted as the neurotoxic species in AD, we show that it is important not to overlook their consequences in other disease contexts. Our findings also provide the rationale to target protein–protein interactions as a novel therapeutic strategy against dementia in which biomolecules such as antibodies or aptamers could be promising candidates.

## Supporting Information Available

Biological replicates for the ThT aggregation assay, negative stain TEM and immunogold labeled TEM images used for quantification, unedited immunoblots, and table of α-synuclein antibody epitopes; supplementary methods for modeling the kinetics of aggregation (PDF)

## Abbreviations

AD: Alzheimer’s disease
PD: Parkinson’s disease
Aβ: amyloid-β
α-syn: α-synuclein
NAC: non-amyloid-β component
ThT: thioflavin T
PK: proteinase K
TEM: transmission electron microscopy
LB: Lewy body
