# Supplementary material for: α-Synuclein Aggregation Is Triggered by Oligomeric Amyloid-β 42 via Heterogeneous Primary Nucleation
Source: J Am Chem Soc. 2023 Aug 9;145(33):18276–85. doi: 10.1021/jacs.3c03212 (PMC10450681; doi:10.1021/jacs.3c03212)
Supplement: Supplementary file 1 — ja3c03212_si_001.pdf [file ja3c03212_si_001.pdf]

Supplementary Information for

α-Synuclein Aggregation is Triggered by Oligomeric Amyloid-β 42 via Heterogeneous Primary Nucleation

Devkee M. Vadukul[1], Marcell Papp[2], Rebecca J. Thrush[1,3], Jielei Wang[1], Yiyun Jin[1], Paolo Arosio[2], and Francesco A. Aprile[1,3]*

[1]Department of Chemistry, Molecular Sciences Research Hub, Imperial College London, London W12 0BZ, UK
[2]Department of Chemistry and Applied Biosciences, Institute for Chemical and Bioengineering, Swiss Federal Institute of Technology, 8093 Zurich, Switzerland
[3]Institute of Chemical Biology, Molecular Sciences Research Hub, Imperial College London, London W12 0BZ, UK

*Author to whom correspondence should be addressed.
Email: f.aprile@imperial.ac.uk , Phone: +44 (0)20 7594 5545

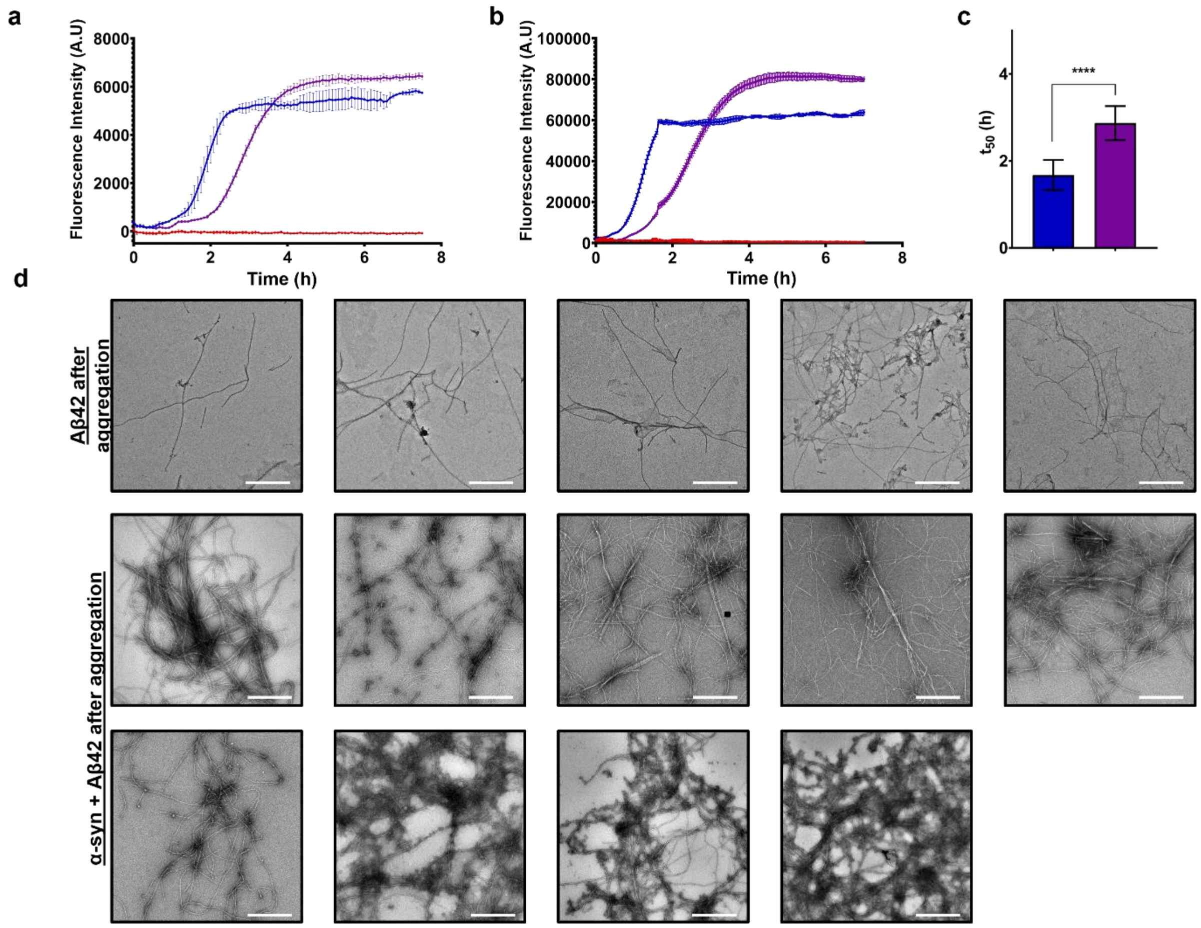

Fig. S1. Aggregation Aβ42 and α-syn aggregated with Aβ42. (a-b) Two independent aggregation assays. α-syn is shown in red, Aβ42 is shown in blue, and α-syn incubated with Aβ42 is shown in purple (c) The t50 of 3 independent aggregation repeats (shown in Fig 1a and Fig S1a-b) were averaged. Error bars are shown as SD. Unpaired, non-parametric Mann Whitey's test, where p = 0.1234 (ns), 0.0332 (*), 0.0021 (**), 0.0002 (***) and <0.0001 (****). (d) Negative stain TEM. All TEM images used to confirm fibril formation of Aβ42 and α-syn incubated with Aβ42 at the end of aggregation. Scale bars are representative of 0.2 µm.

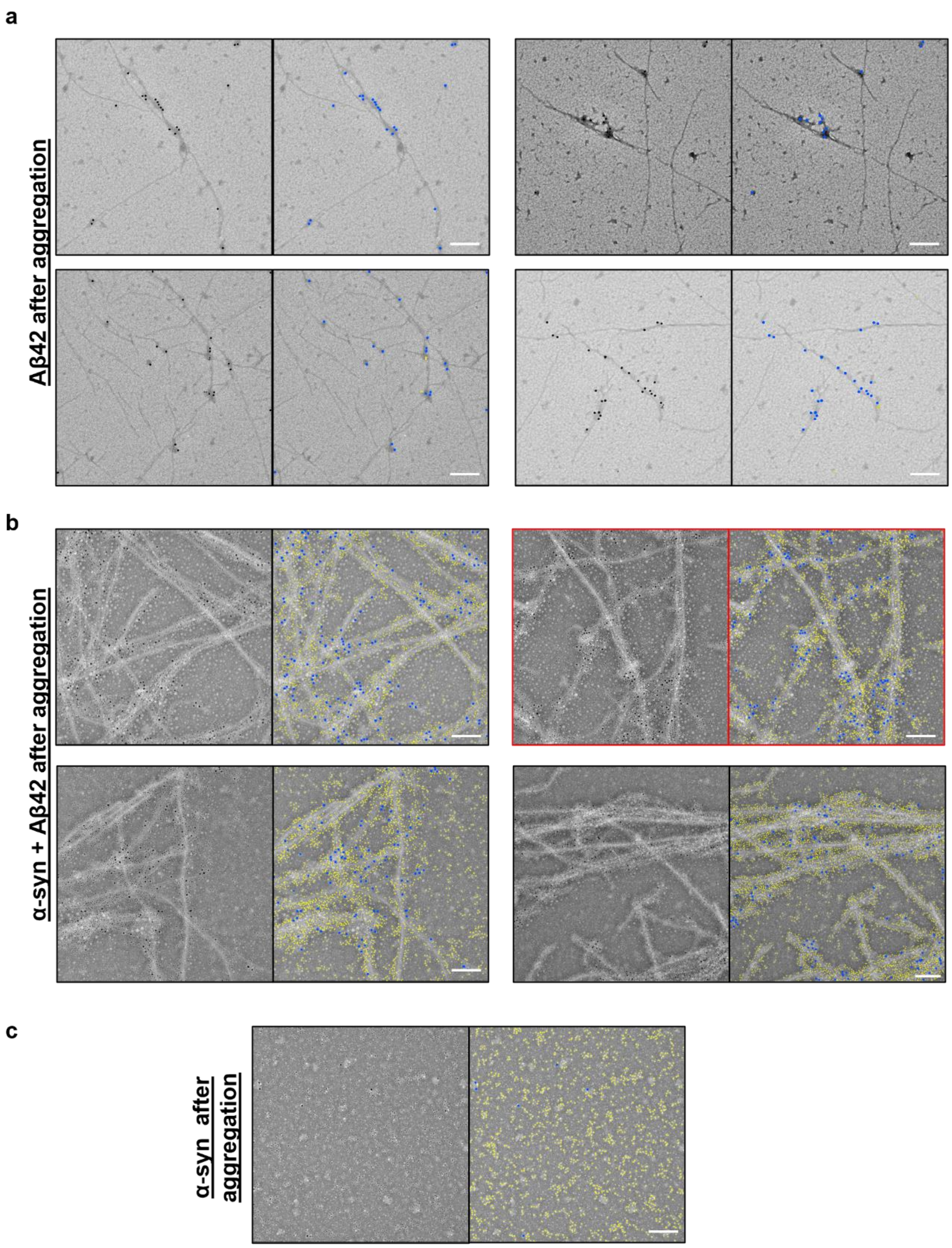

Fig. S2. Immunogold labelling and negative stain TEM. All images used to quantity fibril length of (a) Aβ42 and (b) the coincubation samples after aggregation. Scale bars are representative of 0.2 μm. Left panel images are raw, unprocessed images and right panel images have been processed to assign 6 nm and 10 nm gold particles yellow and blue colors, respectively. Yellow and blue dots are represented as 1.5X their actual size for clarity. (c) α-syn after aggregation is also shown. Yellow 6 nm gold particles bind to the anti-α-syn primary antibody, and blue 10 nm gold particles bind to the anti-Aβ42 6E10 primary antibody.

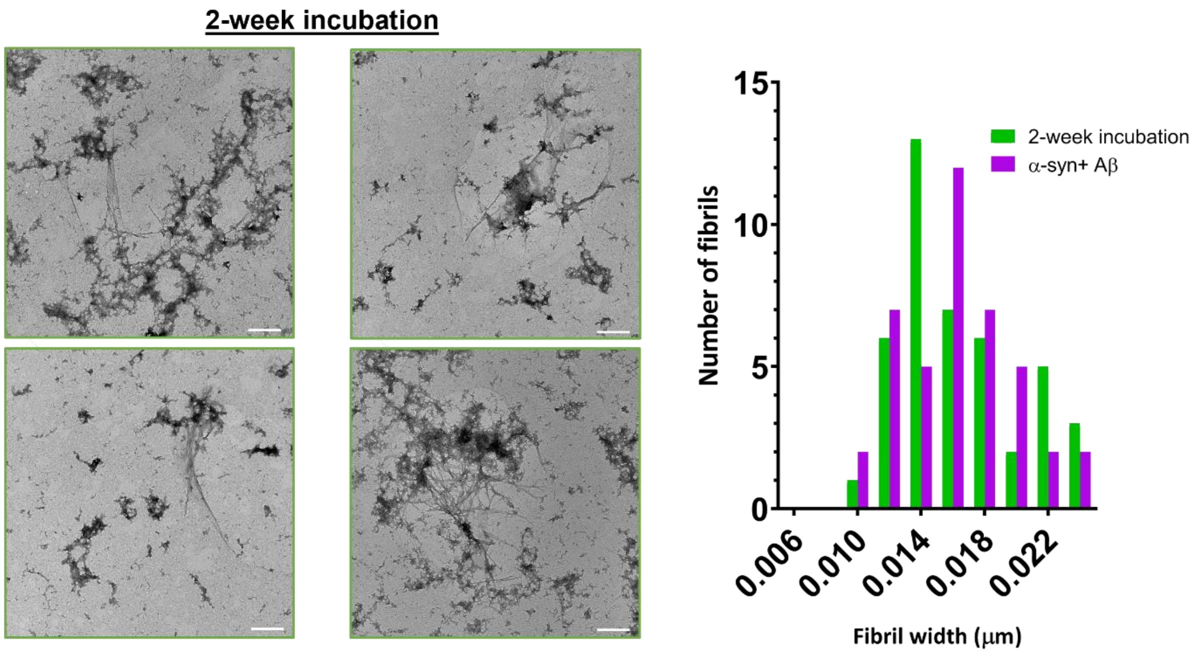

Fig. S3 Negative stain TEM of α-syn fibrils formed in quiescent conditions after 2-week incubation at 37°C and width comparison with coincubation sample. 60 μM α-syn was aggregated under quiescent conditions at 37°C for 2 weeks (2-week incubation, green, n=43). Scale bars are representative of 0.5 μm. We found there was no significant difference in the fibril widths formed in quiescent conditions and of those in the coincubation sample (both 16 nm).

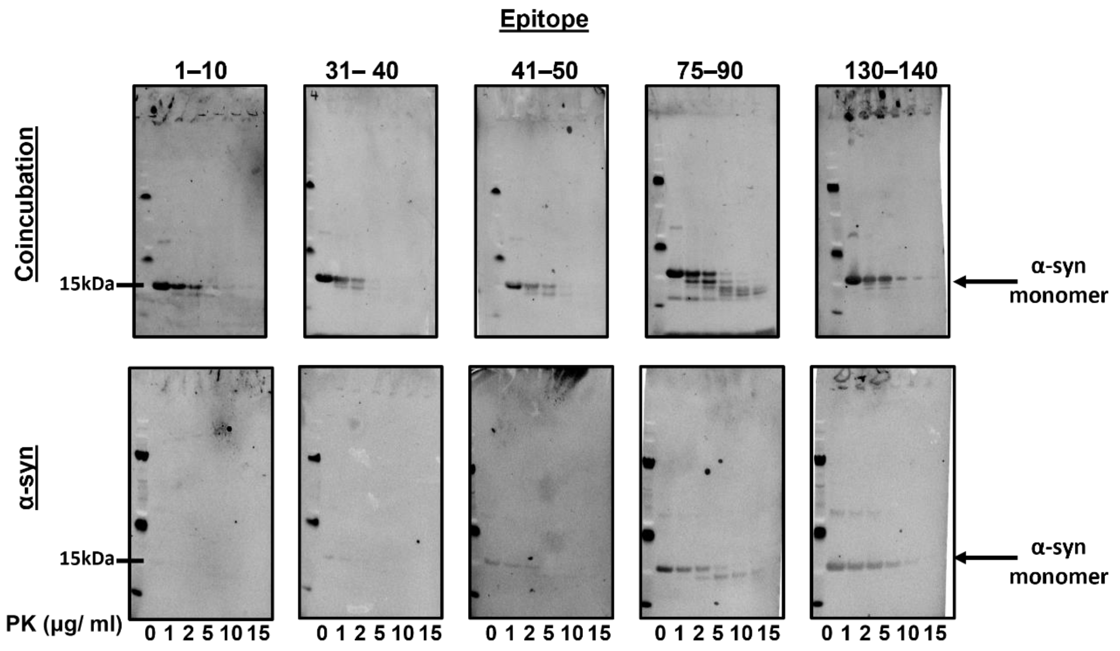

Fig. S4. Proteinase K digestion of α-syn fibrils in the presence and absence of Aβ42. Fibrils were collected at the end of aggregation and treated with increasing concentrations of proteinase K for 20 minutes. SDS-PAGE and western blotting with antibodies scanning the sequence of α-syn (Table S1) were used to assess the PK-stability of fibrils based on the band intensity of the putative monomer.

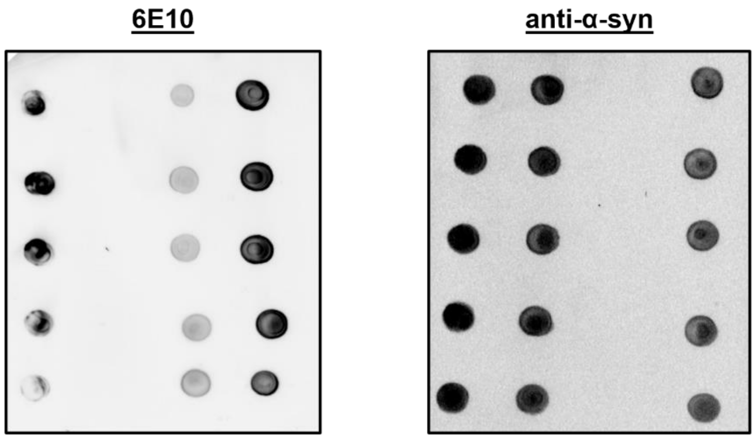

Fig. S5. Solubility of Aβ42 and α-syn after coincubation. Dot blot analysis on the soluble fractions of aggregated samples detected with 6E10 (left) and anti-α-syn (right) primary antibodies. 5 repeats for each sample were quantified (**Fig. 2b**).

a

b

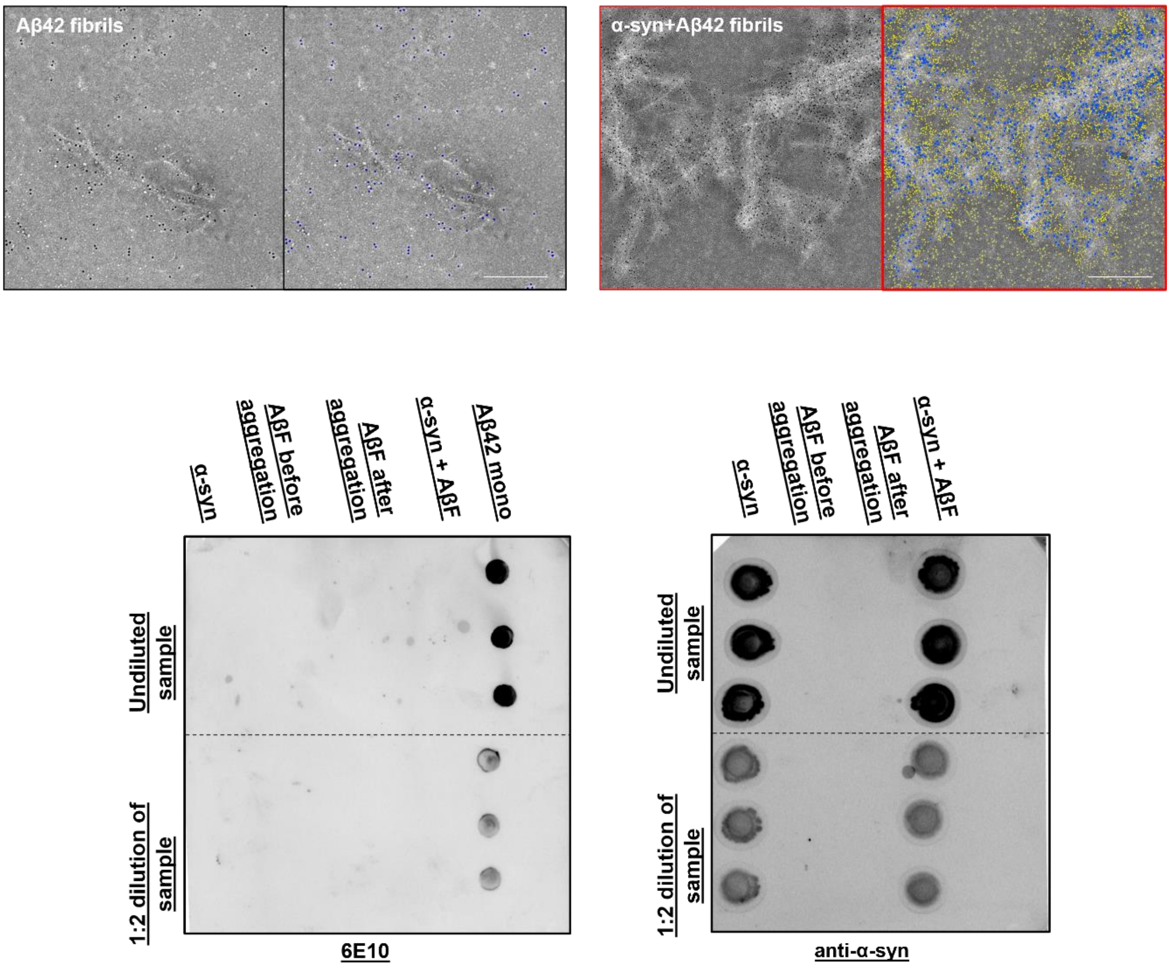

Fig. S6. Aβ42 fibrils do not nucleate the aggregation of α-syn. (a) Immunogold labelling of Aβ fibrils alone at the end point of aggregation and of α-syn incubated with Aβ42 fibrils at the end of aggregation (as shown in Fig 3b, highlighted here in red). In both cases, fibrils are highly decorated with 10 nm gold particles (Ab–6AuNPs, yellow) specific for Aβ42 as opposed to 6 nm gold particles labelling α-syn (Ab–10AuNPs, blue). Left panel images show the raw, unprocessed images and the right panel images have been processed to assign 6 nm and 10 nm gold particles yellow and blue colors, respectively. Yellow and blue dots are represented as 1.5X their actual size for clarity. Scale bars are representative of 0.5 μm (b) Uncropped dot blot analysis shown in Fig. 3d. Dot blot analysis was carried out on the soluble fractions of aggregated samples detected with 6E10 (left) and anti-α-syn (right) primary antibodies. No soluble Aβ42 was detected in any sample except freshly purified Aβ42 monomers as expected and similar intensities of α-syn were detected in the α-syn only and α-syn aggregated with Aβ42 fibrils sample.

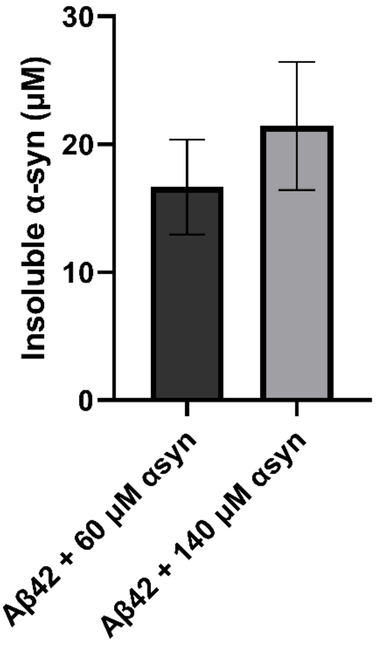

Fig S7. Insoluble α-syn concentration at the end of aggregation (~60 h) when 2 μM Aβ42 was aggregated in the presence of 60 or 140 μM α-syn. Insoluble α-syn concentration was determined by subtracting the soluble α-syn concentration at the end of concentration from the initial monomer concentration. We find there is no significant difference in the concentration of insoluble α-syn between the two conditions (Unpaired t-test).

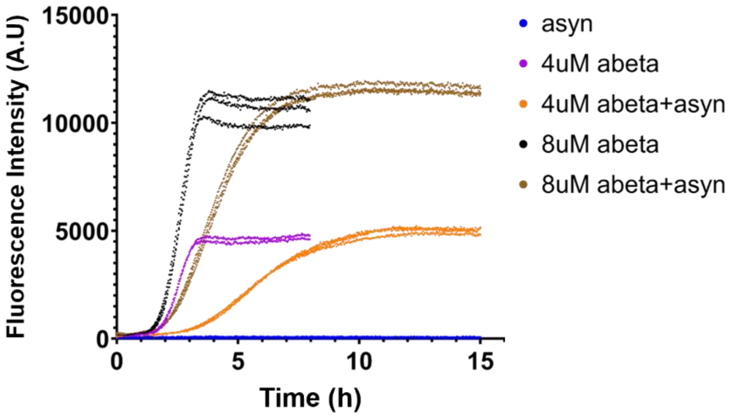

Fig. S8. 140 μM α-syn aggregated with 4 and 8 μM Aβ42. ThT aggregation assay showing that the final fluorescence intensity of the coincubation sample increases as a function of initial Aβ42 concentration.

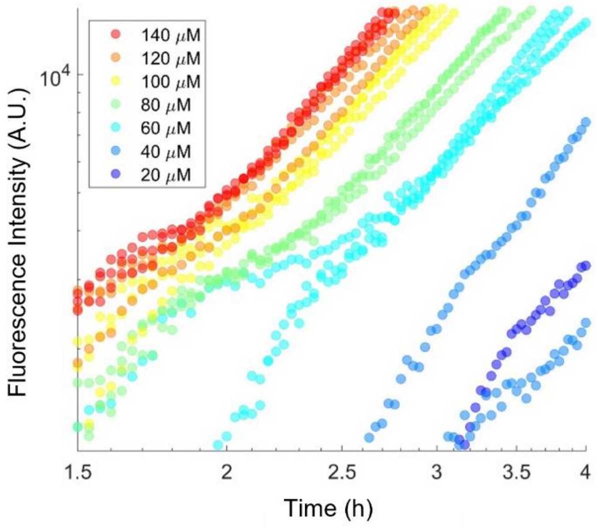

Fig. S9. ThT fluorescence kinetics of the aggregation of α-syn. The fluorescence signal increases linearly on the logarithmic scale plot implying polynomial time evolution of the total fibril mass in the first 4 hours of coincubation of Aβ42 and α-syn.

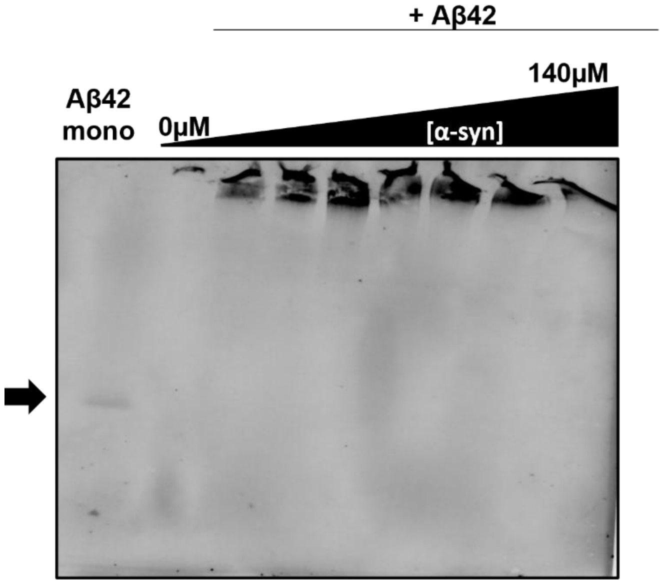

Fig. S10. Aβ42 remains soluble at increasing molar ratios when incubated with α-syn. Native-PAGE and western blot analysis revealed that, in contrast to monomeric Aβ42, after incubation Aβ42 was detected only has aggregates stuck in the well of the gel. In the presence of increasing α-syn concentrations in the range 20-100 μM, Aβ42 remains as soluble high molecular weight assemblies.

Epitope	Sequence
**1-10**	MDVFMKGLSKC
**31-40**	GKTKEGVLYVC
**41-50**	GSKTKEGVVHC
**75-90**	CTAVAQKTVEGAGSIAAA
**130-140**	CEGYQDYEPEA

Table S1. Epitopes of the anti-α-syn antibodies used in the PK digestion analysis shown in Fig.1e.

Supplementary methods

Modelling of aggregation kinetics. We consider the generation of oligomers which can be composed of both Aβ42 and α-syn. α-syn monomers then bind to the surface of oligomers where they form fibril-competent nuclei. We applied a kinetic model previously developed to derive molecular mechanisms of aggregation from macroscopic kinetic profiles.[1-4] Specifically, we considered the model described in Galvagnion et al.,[5] and derived differential rate equations describing the number concentration of oligomers O (eq. (1)) and α-syn fibrils F (eq. (3)) as well as the fibril mass concentration M (eq. (4)). The rate of oligomer formation can be described by eq. (1):

$$\frac{dO}{dt} = k_o \cdot m_{A\beta 42}^{o_1} \cdot m_{\alpha syn}^{o_2} - k_d \cdot O \qquad (1)$$

where $m_{A\beta 42}$ and $m_{\alpha syn}$ are the concentration of monomeric Aβ42 and α-syn, o_1 and o_2 are the apparent reaction order of oligomer formation relative to monomer concentration of Aβ42 and α-syn, respectively, k_o and k_d denote the rate constant of oligomer formation and dissociation. We note that considering homo-oligomerization of Aβ42 is a special case of eq. (1) where $o_2 = 0$. Although in principle the dissociation of oligomers may be a function of their surface coverage Φ, the large excess of a-syn even in the lowest α-syn-Aβ42 ratio suggests that the coverage remains close to 1 at all α-syn concentrations. Hence, k_d is assumed constant in our kinetic model.
In the early time limit $m_{A\beta 42}$ and $m_{\alpha syn}$ is approximated by their initial concentrations, and hence eq. (1) can be solved analytically for O:

$$O = \frac{k_o}{k_d} \cdot m_{A\beta 42}^{o_1} \cdot m_{\alpha syn}^{o_2} \cdot (1 - e^{-k_d t}) \qquad (2)$$

We hypothesize that binding of monomeric α-syn happens on a much shorter time scale then the other processes, hence binding equilibrium is reached instantaneously. Moreover, due to the large excess of α-syn compared to Aβ42 saturated surface coverage is assumed on the oligomers, that is, $\Phi \approx 1$ in all cases. Subsequent to binding, heterogeneous primary nucleation occurs on the surface of oligomers. The rate of nuclei formation is written as:

$$\frac{dP}{dt} = k_n \cdot O \cdot a \cdot \Phi \cdot m_{\alpha syn}^{n} = k_n \cdot a \cdot \Phi \cdot m_{\alpha syn}^{n} \cdot \frac{k_o}{k_d} \cdot m_{A\beta 42}^{o_1} \cdot m_{\alpha syn}^{o_2} \cdot (1 - e^{-k_d t}) \qquad (3)$$

a denotes the number of binding sites of α-syn on the surface of one oligomer, n is the apparent reaction order of nucleation relative to monomeric α-syn. Since secondary processes were assumed to be negligible at these conditions, substituting eq. (2) into eq. (3), and subsequent integration yields:

$$P = \frac{k_o k_n}{k_d} \cdot \mathrm{a} \cdot \Phi \cdot m_{A\beta 42}^{o_1} \cdot m_{\alpha syn}^{o_2+n} \cdot \left(t - \frac{1}{k_d}(1 - e^{-k_d t})\right) \qquad (4)$$

The fibril mass increases over time via elongation:

$$\frac{dM}{dt} = k_+ \cdot K_M \cdot \frac{m_{\alpha syn}}{K_M + m_{\alpha syn}} \cdot P \qquad (5)$$

Integration of eq. (5) provides the total fibril mass at time point t:

$$M = \frac{k_+ k_n k_o}{k_d} \cdot K_M \cdot a \cdot \Phi \cdot m_{A\beta 42}^{o_1} \cdot \frac{m_{\alpha syn}^{o_2+n+1}}{K_M + m_{\alpha syn}} \cdot \left(\frac{t^2}{2} - \frac{t}{k_d} + \frac{1}{k_d^2} \cdot (1 - e^{-k_d t})\right) \qquad (6)$$

Which can be further simplified upon expansion of the exponential expression into:

$$M = \frac{1}{6} k_+ k_n k_o \cdot K_M \cdot a \cdot \Phi \cdot m_{A\beta 42}^{o_1} \cdot \frac{m_{\alpha syn}^{o_2+n+1}}{K_M + m_{\alpha syn}} \cdot t^3 \qquad (7)$$

Assuming linear dependence of the measured fluorescence intensity on the fibril mass concentration ($F.I. = \lambda \cdot M$) we get:

$$F.I. = C \cdot m_{A\beta 42}^{o_1} \cdot \frac{m_{\alpha syn}^{o_2+n+1}}{K_M + m_{\alpha syn}} \cdot t^3 \quad (8)$$

Where C, $(o_2 + n + 1)$ and K_M were evaluated by a global fit to the experimental ThT profiles curves by MATLAB homemade programs.

Supplementary References

(1) Cohen, S. I.; Vendruscolo, M.; Welland, M. E.; Dobson, C. M.; Terentjev, E. M.; Knowles, T. P. Nucleated polymerization with secondary pathways. I. Time evolution of the principal moments. *J Chem Phys* **2011**, *135* (6), 065105. DOI: 10.1063/1.3608916.
(2) Cohen, S. I.; Vendruscolo, M.; Dobson, C. M.; Knowles, T. P. From macroscopic measurements to microscopic mechanisms of protein aggregation. *J Mol Biol* **2012**, *421* (2-3), 160-171. DOI: 10.1016/j.jmb.2012.02.031.
(3) Michaels, T. C. T.; Saric, A.; Curk, S.; Bernfur, K.; Arosio, P.; Meisl, G.; Dear, A. J.; Cohen, S. I. A.; Dobson, C. M.; Vendruscolo, M.; et al. Dynamics of oligomer populations formed during the aggregation of Alzheimer's Abeta42 peptide. *Nat Chem* **2020**, *12* (5), 445-451. DOI: 10.1038/s41557-020-0452-1.
(4) Cohen, S. I.; Linse, S.; Luheshi, L. M.; Hellstrand, E.; White, D. A.; Rajah, L.; Otzen, D. E.; Vendruscolo, M.; Dobson, C. M.; Knowles, T. P. Proliferation of amyloid-beta42 aggregates occurs through a secondary nucleation mechanism. *Proc Natl Acad Sci U S A* **2013**, *110* (24), 9758-9763. DOI: 10.1073/pnas.1218402110.
(5) Galvagnion, C.; Buell, A. K.; Meisl, G.; Michaels, T. C.; Vendruscolo, M.; Knowles, T. P.; Dobson, C. M. Lipid vesicles trigger alpha-synuclein aggregation by stimulating primary nucleation. *Nat Chem Biol* **2015**, *11* (3), 229-234. DOI: 10.1038/nchembio.1750.
